# Estimating the self‐thinning boundary line for oak mixed forests in central China by using stochastic frontier analysis and a proposed variable density model

**DOI:** 10.1002/ece3.9064

**Published:** 2022-09-20

**Authors:** Shisheng Long, Siqi Zeng, Zhenwei Shi, Shengyang Yang

**Affiliations:** ^1^ Faculty of Forestry Central South University of Forestry and Technology Changsha China

**Keywords:** dominant height, quantile regression, Simpson index, stochastic frontier analysis, variable density

## Abstract

A suitable self‐thinning model is fundamental to effective density control and management. Using data from 265 plot measurements in oak mixed forests in central China, we demonstrated how to estimate a suitable self‐thinning line for oak mixed forests from three aspects, i.e., self‐thinning models (Reineke's model and the variable density model), statistical methods (quantile regression and stochastic frontier analysis), and the variables affecting stands (topography and stand structure factors). The proposed variable density model, which is based on the quadratic mean diameter and dominant height, exhibited a better goodness of fit and biological relevance than Reineke's model for modeling the self‐thinning line for mixed oak forests. In addition, the normal‐truncated normal stochastic frontier model was superior to quantile regression for modeling the self‐thinning line. The altitude, Simpson index, and dominant height–diameter ratio ($H_{d}$/*D*) also had significant effects on the density of mixed forests. Overall, a variable density self‐thinning model may be constructed using stochastic frontier analysis for oak mixed forests while considering the effects of site quality and stand structure on density. The findings may contribute to a more accurate density management map for mixed forests.

## Graphical abstract

The proposed variable density model, which is based on the quadratic mean diameter and dominant height, showed a better goodness of fit, residual distribution and biological significance.The normal‐half normal stochastic frontier model was superior to quantile regression in modeling the self‐thinning line

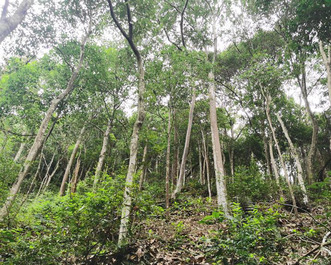

## 1 INTRODUCTION

**FIGURE 1:**
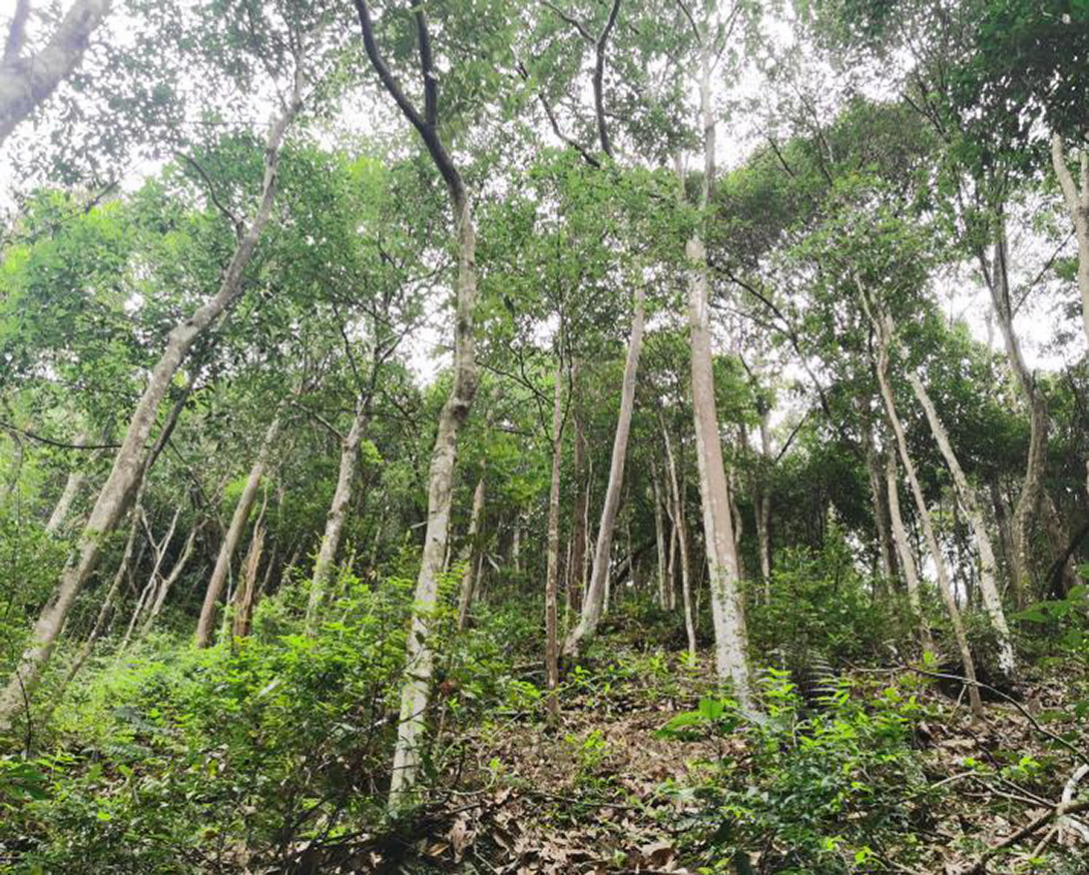
The status of oak mixed forest in central China

Oak is one of the main species of broad‐leaved forests in subtropical, tropical, and temperate regions (Nixon, 1993; Perea et al., 2017). In China, oak occupies a large proportion of natural forests. The 8th Chinese National Forest Inventory shows that the total area of *Quercus* in China is 16.72 million hectares. The area and accumulation of oaks account for 10.15% and 12.94%, respectively, of the national forest (Li et al., 2001; Wang et al., 2019). However, oak forests in China face quality problems related to an excessive stand density, curved trunks, and incomplete crowns as a result of serious damage (Figure 1). At present, the average volume per hectare of oak forests in China is approximately 77.39 m^3^, while in Germany, it is 305 m^3^ per hectare (Hou et al., 2017). Low‐quality oak forests seriously affect overall forest quality and resources in oak distribution areas and impact regional ecological functions. Therefore, understanding the stand density structure, growth, and management of oak forests is particularly important and urgent.

Self‐thinning in forests is caused by limited resources, including light and growing space (Pretzsch & Biber, 2005). The number of trees decreases when the available resources cannot meet the normal demands of growing trees (Vospernik & Sterba, 2015; Yang & Burkhart, 2017). Reineke's stand density index is based on a linear relationship between the logarithm of the stand density and the logarithm of the mean diameter, and the slope of the linear model is −1.605 (Reineke, 1933). The −3/2 rule outlined by Yoda et al. (1963) relates the mean plant volume (or biomass) to the number of plants per unit area. Other density indexes, such as those presented by Hart (1926) and West et al. (1997), have also received widespread attention. These rules are scientifically meaningful for forestry research. However, several studies have challenged these conclusions because the slope did not equal −1.605 or −3/2 in all studies (Gadow, 1986; Westoby & Howell, 1986; Zeide, 1985). Moreover, the maximum size–density relationship (MSDR) under different environmental conditions or tree species can differ greatly (Charru et al., 2012; Vospernik & Sterba, 2015). Despite remarkable advances, the self‐thinning rule continues to be a controversial issue, and there are still many problems to solve. Currently, three aspects should be considered when estimating the self‐thinning line of a tree species: a self‐thinning model, statistical methods, and the variables that affect the MSDR.

**FIGURE 2:**
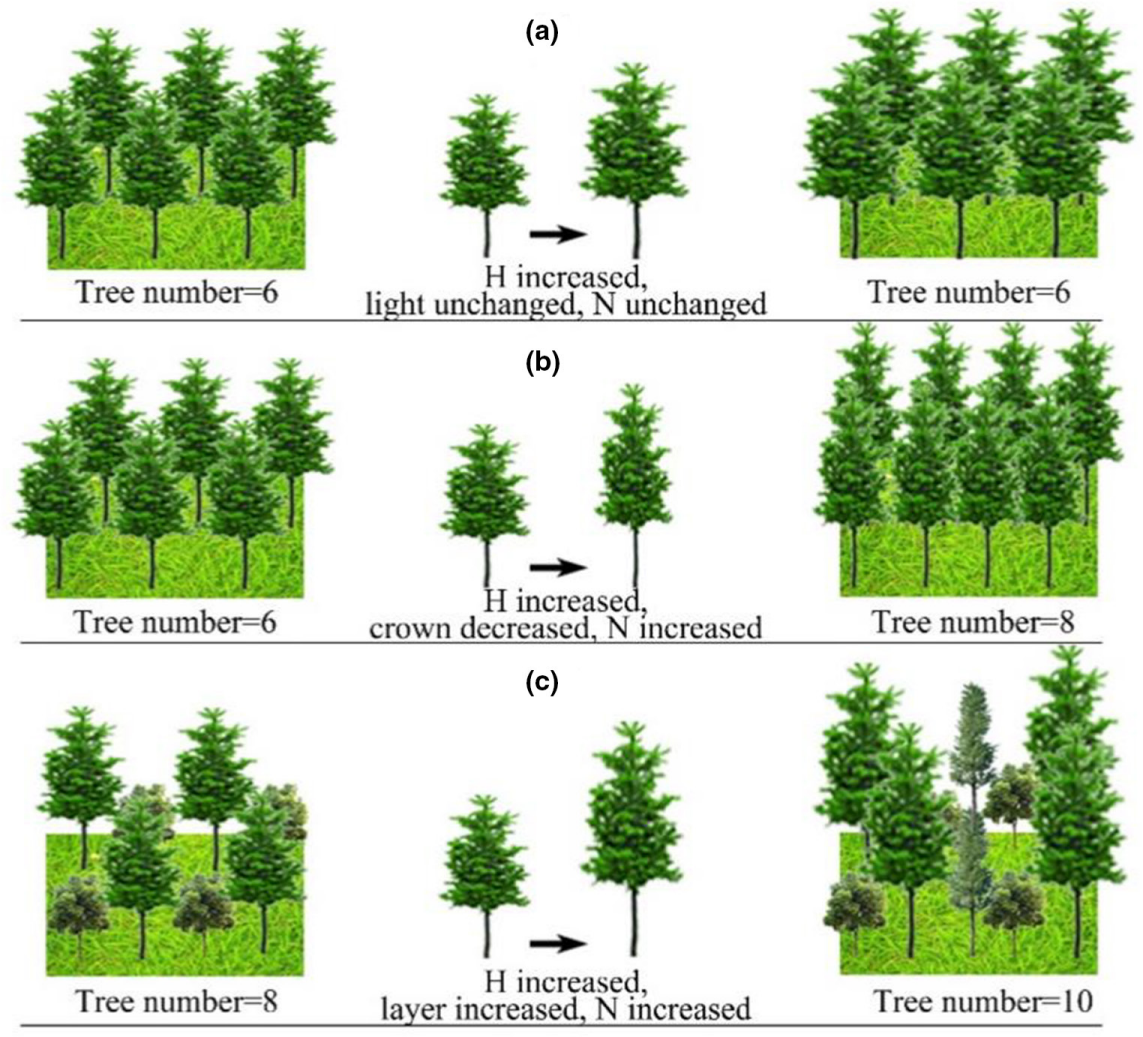
Three cases for the effect of tree height on stand density (*H* is the dominant height, *N* is the stand density)

In general, self‐thinning models are built with stand density as the dependent variable and tree size (such as the mean diameter, mean height, or mean biomass) as the independent variable (Hart, 1926; Reineke, 1933; Yoda et al., 1963). After analyzing the maximum size density using reasoning and empirical evidence, Zeide (1985, 1987, 1991) and Burkhart (2013) concluded that measurements based on the diameter are preferable. The number of trees was most closely related to the average crown, but the height and tree volume were not as highly correlated with the crown width as the average diameter. Zeide (2005) further specified that an increase in tree height did not affect the stand density because trees grow side‐by‐side rather than on top of each other. The average height of a stand does not change the amount of light available to the trees growing in a given area; it only lifts the crowns without securing additional light per unit area (Figure 2a). However, the conclusions presented by Zeide (2005) were based on plantation forests, and it is not clear if the conclusions are applicable to mixed forests because the characteristics of diameter and height were significantly different from those of plantation forests. It is possible that the effects of height on the density in mixed forests are different from those in plantation forests in the following two aspects.

1. Tree height influences the size of the crown, and the crown influences the stand density.

When other factors (such as the diameter and wood quality) are equal, the correlation between the height and the crown dimensions is negative (Briegleb, 1952; Harding & Grigal, 1985; Ouellet, 1985). This means that taller trees must have smaller crowns than shorter trees with the same diameter (Zeide, 2005). As shown in Figure 2b, when the diameter of tree i is kept constant, an increased height will lead to a decrease in the crown width. Then, the canopy overlaps and competition is reduced in the stand, which may lead to a decrease in the death rate. Moreover, the growth space occupied in the horizontal dimension will be reduced for each tree, and the number of trees per unit area may increase, leading to an increase in the stand density.

2 The dominant height influences the number of canopy levels, and the number of canopy levels influences the tree number.

Unlike a single canopy level in plantation forests, two or more canopy levels are often found in mixed forests. As shown in Figure 2c, only two canopy levels were found in the mixed forests when the dominant height was low, and the tree number with the maximum stand carrying capacity was 8. The number of canopy levels may increase when the dominant height increases, and then the growth space occupied in the vertical dimension will be enlarged, which leads to an increase in the stand density (e.g., the tree number increases to 10).

Applying different statistical methods leads to different conclusions regarding the validity of the self‐thinning model. In previous studies, ordinary least squares (OLS) were frequently used for fitting the self‐thinning model, and its validity relies on the data points reflecting the MSDR. Four methods for selecting data points have been proposed: the visual method (Osawa & Allen, 1993; Osawa & Sugita, 1989), quantifications of mortalities in successive measurements (Westoby, 1984), the selection of points from each equal‐width interval (Bi & Turvey, 1997; Newton, 2006) and the relative density method (Solomon & Zhang, 2002). However, such methods still harbor a degree of subjectivity and may lead to inaccurate results due to a lack of experience. Fortunately, with the advancement of modern statistical methods, the self‐thinning line can be fitted using principal component analysis (Weller, 1987), stochastic frontier analysis (Bi et al., 2000; Charru et al., 2012), and linear quantile regression (Vospernik & Sterba, 2015; Zhang et al., 2013). Due to their favorable inferential properties, stochastic frontier analysis and quantile regression are widely considered to be the current state‐of‐the‐art methods for estimating the self‐thinning line (Andrews et al., 2018; Bi, 2001; Kimsey et al., 2019). The results of previous studies favored stochastic frontier analysis, although quantile regression performed nearly as well in some cases (Salas‐Eljatib & Weiskittel, 2018). Accordingly, it is necessary to further explore and compare the two methods in terms of suitability and validity, especially for mixed forests of specific species.

Studies have shown that the MSDR influences tree species, site quality, nutrient availability, climate, and other factors, which is why the intercept and slope of the self‐thinning line change (Comeau et al., 2010; Harper, 1977). In general, shade‐tolerant species show a higher “stock ability” than shade‐intolerant species (Charru et al., 2012; Pretzsch & Biber, 2005). Stand origin has been shown to affect the slope of the self‐thinning line, with planted stands having a less steep slope (Charru et al., 2012; Weiskittel et al., 2009). The soil nutrient regime has a positive effect on the intercept and a negative effect on the slope of the self‐thinning line (Reyes‐Hernandez et al., 2013; Weiskittel et al., 2009). The climate has also been shown to have a significant influence on the maximum stand carrying capacity (Aguirre et al., 2018; Condés et al., 2017; de Prado et al., 2020). Most studies have focused on the influence of habitat factors (e.g., site quality, climate, tree species diversity) on the MSDR, but less attention has been given to stand structure factors (e.g., ratio of height to diameter) (Ducey et al., 2017; Salas‐Eljatib & Weiskittel, 2018).

The objective of this study was to explore how to estimate a suitable self‐thinning line for mixed oak forests from three aspects: a self‐thinning model, a statistical method, and the influential variables. Therefore, we aimed to: (1) propose a new self‐thinning model and investigate the suitability of the proposed model for oak mixed forests through a comparison with Reineke's model; (2) evaluate the statistical approach in fitting self‐thinning relationships in mixed forests; and (3) relate the stand maximum density to topography and stand structure factors.

## 2 MATERIALS AND METHODS

### 2.1 Study site and data

**FIGURE 3:**
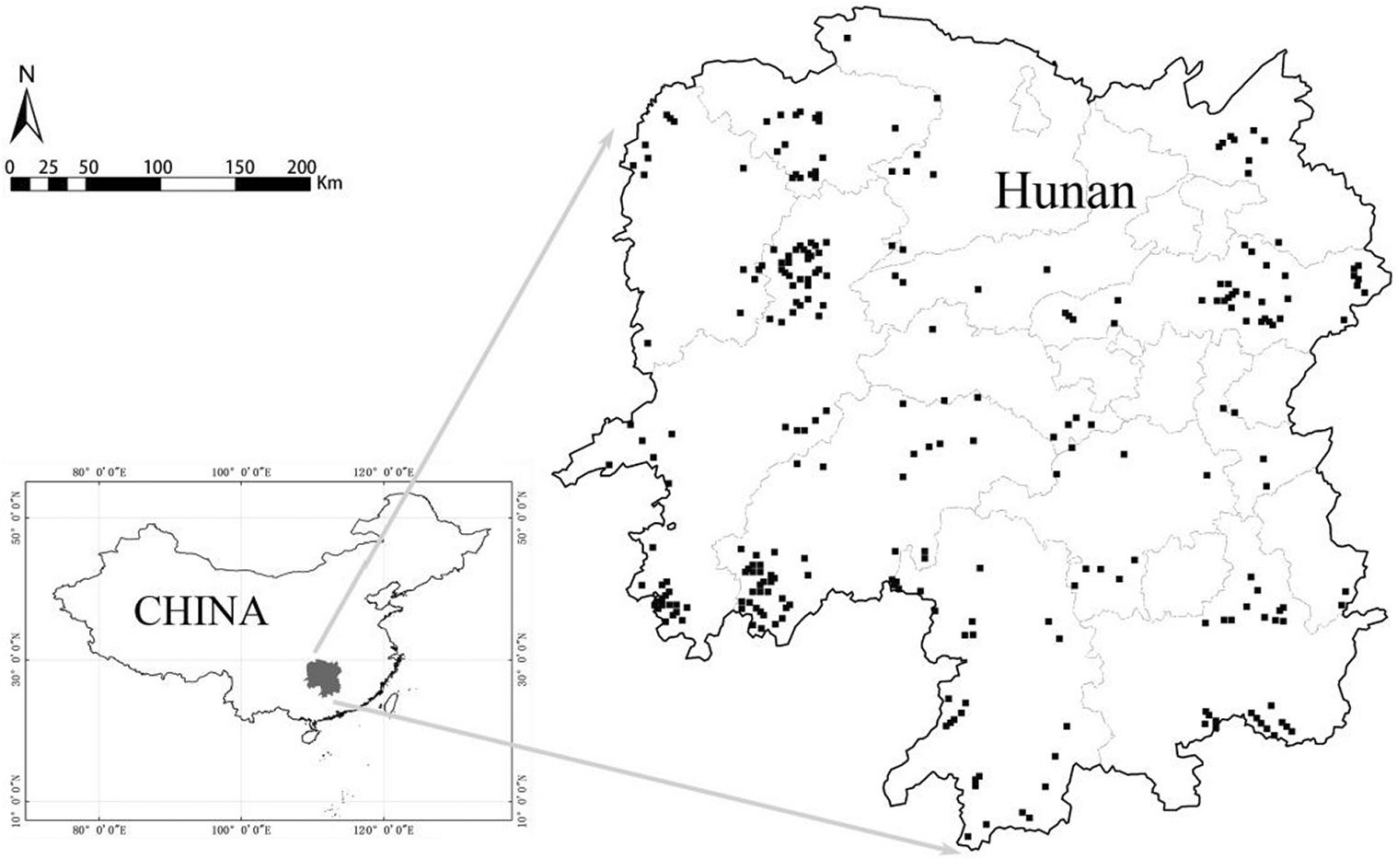
Map showing Hunan Province, central China. Each black point represents one of the sample plots.

The study site is located in Hunan Province, central China, with longitude and latitude ranges of 108°47′–114°15′E and 24°38′–30°08′N, respectively (Figure 3). The study area has an altitude range from 24 m to 2122 m and complex landforms, including hills, flatlands, and mountains. This area has a typical continental subtropical monsoon humid climate with a wet spring and summer and less rain in the autumn and winter, i.e., characteristics of drought. The mean annual temperature is 15–18°C, and the mean annual precipitation is 1200–1700 mm. The study area is dominated by red soil and yellow soil with a small amount of calcareous soil and tidal soil. Hunan Province is located in the central subtropical evergreen broad‐leaved forest zone. The main tree species are *Cunninghamia lanceolata*, *Pinus massoniana*, *Liquidambar formosana*, *Cinnamomum camphora,* and *Cyclobalanopsis glauca*, and the main oak species are *Cyclobalanopsis glauca*, *Lithocarpus glaber*, *Castanopsis eyrei,* and *Quercus chenii*.

After statistical analysis and data collection, 265 sample plots were selected from the 8th Chinese National Forest Inventory (Figure 3). Sample plots were surveyed in 2004 and 2014, and their size was 25.82 × 25.82 m. To ensure the consistency and representativeness of the research samples, each sample plot was selected based on the following criteria: (1) the canopy density was higher than 0.60; (2) the number of trees per hectare exceeded 500; and (3) the oak species accounted for more than 15% of all trees in the sample plot. Within each plot, the diameter at breast height (DBH) (measured using a diameter tape, minimum recording limit is 5 cm) and dominant height (arithmetic mean of seven dominant trees in the main canopy layer, measured using a Blume–Leiss hypsometer) were measured, and the tree species, trees per hectare, plot position (*X* and *Y* coordinates), elevation, slope, aspect, slope position, and soil were recorded (measured using a global positioning system and compass). The dataset was randomly split into two parts: 70% (185 plots) for modeling and 30% (80 plots) for validation. Information on the plots is shown in Table 1.

**TABLE 1:** Main characteristics of the plots in 2004 (mortality was calculated as the number of dead trees in 2004–2014 divided by the total number in 2004 and QMD is the quadratic mean diameter)

Variables of plots in 2004	Mean	Min	Max	SD
Mean QMD (cm)	11.2	6.9	23.1	3.0
Dominant height (m)	11.9	6.2	16.4	2.2
Basal area (m^2^·ha^−1^)	3.25	13.4	57.3	6.9
Trees per hectare (trees·ha^−1^)	1506	510	3690	622
Mortality (%)	35.49	1.30	69.23	17.04
Proportion of oak (%)	37.37	15.09	81.03	18.72
Altitude (m)	546	43	1470	329
Slope (°)	30	0	50	10

### 2.2 Self‐thinning model

#### 2.2.1 Reineke's model (RM)

Reineke's stand density index was used in this study to fit the self‐thinning line.

(1) $$\ln\;N=\varphi_{1}+\varphi_{2}\;\ln\;D_{g}$$

where *N* is the number of trees per hectare, $D_{g}$ is the quadratic mean diameter, $\varphi_{2}$ is the species‐specific slope, $\varphi_{1}$ is the species‐specific intercept, and ln is the natural logarithm.

#### 2.2.2 Variable density model (VDM)

Yoda et al. (1963) proposed the relationship between the mean tree weight or the mean stem volume *w* and the stand density *N* in fully stocked pure stands during the self‐thinning process as

(2) $$w=k_{1}N^{a}$$

where $k_{1}$ and *a* is the species‐specific parameter.

According to its formulation, *w* is a power function of the DBH and height:

(3) $$w=k_{2}D^{b}H^{c}$$

For Equation (2) and Equation (3),

(4) $$w=k_{1}N^{a}=k_{2}D^{b}H^{c}$$

Equation (4) means that *N* can also be viewed as a function of the DBH and height:

(5) $$N={\left[\left(k_{2}/k_{1}\right)D^{b}H^{c}\right]}^{1/a}$$

where $k_{1}$, $k_{2}$, *a*, *b*, and *c* are constants. Let $k_{4}={\left(k_{2}/k_{1}\right)}^{1/a}$, α=b/a, and β=c/a. Equation (5) can then be simplified to Equation (6).

(6) $$N=k_{4}D^{b_{1}}H^{b_{2}}$$

Taking the natural logarithm of each side of Equation (6),

(7) $$\ln\;N=k+\alpha \;\ln\;D+\beta \;\ln\;H_{d}$$

Equation (7) can be viewed as a variable density model to fit the self‐thinning line for mixed forests, where α and β are the species‐specific slopes, *k* is the species‐specific intercept, *D* is the quadratic mean diameter, and $H_{d}$ is the dominant height.

### 2.3 Statistical approach to fit the self‐thinning line

#### 2.3.1 Quantile regression

Compared with conventional statistical methods (such as OLS and PCA), quantile regression, which was introduced by Koenker and Basset (1978), is more flexible and provides parameter estimates for linear regressions fit to any portion (i.e., quantile) of a response distribution, including estimates near the observed bounds of the distributions, without imposing stringent assumptions on the distribution of the error (Koenker & Hallock, 2001). The parameter of the τth quantile model can be estimated by the asymmetric loss function that minimizes the absolute value of residuals, as shown in Equation (8)

(8) $$\min_{\gamma }\left[\sum_{\left\{i\left|y_{i}\geq x_{i}\gamma \right.\right\}}\tau \left|y_{i}-x_{i}\gamma \right|+\sum_{\left\{i\left|y_{i}<x_{i}\gamma \right.\right\}}\left(1-\tau \right)\left|y_{i}-x_{i}\gamma \right|\right]$$

where $y_{i}$ is the vector of the dependent variables, $x_{i}$ is the vector of the independent variables, τ is a predetermined quantile between 0 and 1, and γ is the coefficient vector, which varies for different τ values (Scharf et al., 1998). In general, τ is 0.900, 0.950, or 0.990 for the self‐thinning line in most studies. In this study, five values of τ (0.900, 0.925, 0.950, 0.975, and 0.990) were used in the quantile regression for estimating the self‐thinning line.

#### 2.3.2 Stochastic frontier analysis

Stochastic frontier analysis has been proposed as an effective and powerful statistical technique that tests for the effect of covariates while allowing the assumption of constant error variance to be relaxed (heteroscedasticity) when all available data are used (Bi et al., 2000; Weiskittel et al., 2009). The original form of the equation is as follows:

(9) $$Y_{i}=AX_{1}^{\beta_{1}}X_{2}^{\beta_{2}}\cdots X_{k}^{\beta_{k}}e^{v_{i}}e^{u_{i}}$$

where $Y_{i}$ is the i‐th observation, $X_{k}$ is the observation of variables, *A* and $\beta_{k}$ are the predictor coefficients, and $e^{v_{i}}$ and $e^{u_{i}}$ are model errors. Taking the natural logarithm of each side of Equation (9),

(10) $$\ln Y_{i}=\ln A+\sum \beta_{k}X_{k}+\varepsilon_{i}$$

where the composed error term $\varepsilon_{i}=v_{i}-u_{i}$ is a compound random variable with two components: $v_{i}$ denotes the random error terms obeying a normal distribution, and $u_{i}$ embodies the one‐side (asymmetric) pair of the composed error term $\varepsilon_{i}$. Several specifications have been considered for $u_{i}$, including a half‐normal distribution, an exponential distribution, and a truncated normal distribution. The two error distributions were combined into three stochastic frontier models in the study, namely normal–half normal (NH), normal–exponential (NE) and normal–truncated normal (NT). All analyses were performed using StataSE version 15. During analysis, the data used in quantile regression and random frontier analysis are only from 2004. And the mortality applied in traditional methods is the incremental data from 2004 to 2014, which are static data.

#### 2.3.3 Comparative performances of the two models or methods

The performances of quantile regression and stochastic frontier analysis were compared using the maximum stand density index (SDI_max_), that is, the maximum number of trees at a given reference average individual size (diameter or height) that can exist in a self‐thinning population (Husch et al., 1972). In general, the larger the ratio of actual stand density to SDI_max_ is, the more crowded the stand and the higher the tree mortality. For Reineke's model, the SDI^'^_max_ is predicted as follows:

(11) $${{\mathrm{SDI}}^{\prime }}_{\max}=N{\left(\frac{D_{g}}{D_{0}}\right)}^{\alpha }$$

For the variable density model, the SDI_max_ is predicted as follows:

(12) $${{\mathrm{SDI}}^{\prime }}_{\max}=N{\left(\frac{D_{g}}{D_{0}}\right)}^{\alpha }{\left(\frac{H_{g}}{H_{0}}\right)}^{\beta }$$

where ${\mathrm{SDI}}_{\max}$ an SDI^'^_max_ are the predicted maximum density at a base‐average tree size, *N* is the actual stand density, $D_{g}$ is the actual stand diameter, $H_{g}$ is the realistic stand dominant height, $D_{0}$ is the standard stand diameter (the value is 16 cm), and $H_{0}$ is the standard stand dominant height (the value is 12 m).

The performances of Reineke's model and the variable density model were compared using the Akaike information criterion (AIC) and the likelihood ratio test (LRT).

### 2.4 Topography and stand structure factors

One aim of this study was to explore the relationship between SDI_max_ and topography and stand structure factors in mixed forests. We fit several models of the form as follows:

(13) $${\mathrm{SDI}}_{\max}=f\left(\theta ,X_{i}\right)+e_{i}$$

where $X_{i}$ is the predictor variables matrix, $f\left(\right)$ is a linear or nonlinear function, θ is a parameter vector of the model, and $e_{i}$ is the random error term that follows a Gaussian distribution with zero mean and variance $\sigma_{e}^{2}$.

The topographic variables included altitude and slope. The stand structure factors included the Shannon index ($H^{\prime }$, nondimensional), Simpson index ($D_{S}$, nondimensional), coefficient of variation of the tree diameter (CV), and height–diameter ratio ($H_{d}$/*D*). $H^{\prime }$ is the Shannon index for species diversity in (14), and $D_{S}$ evaluates the probability that two individuals chosen at random belong to the same species (15). CV is defined as the ratio of the DBH standard deviation divided by the mean DBH in each cell. $H_{d}$/*D* is defined as the ratio of the dominant height (m) divided by the mean DBH (m).

(14) $$H^{\prime }=-\sum_{i=1}^{S}P_{i}\ln P_{i}$$

where S is the number of tree species, $P_{i}$ is the proportion of the total sample belonging to the *i*th tree species, and ln is the natural log.

(15) $$D_{S}=1-\frac{\sum n\left(n-1\right)}{N\left(N-1\right)}$$

where n is the total number of specific tree species and N is the total number of all tree species. The information of the variables is shown in Table 2.

**TABLE 2:** Main characteristics of the four stand structure factors

Factors	Mean	Min	Max	SD
$H^{\prime }$	1.41	0.36	2.26	0.47
$D_{S}$	0.64	0.18	0.87	0.19
CV	0.47	0.26	0.79	0.12
$H_{d}$/D	121.76	55.95	216.60	34.04

## 3 RESULTS

### 3.1 Evaluation of the self‐thinning model

**FIGURE 4:**
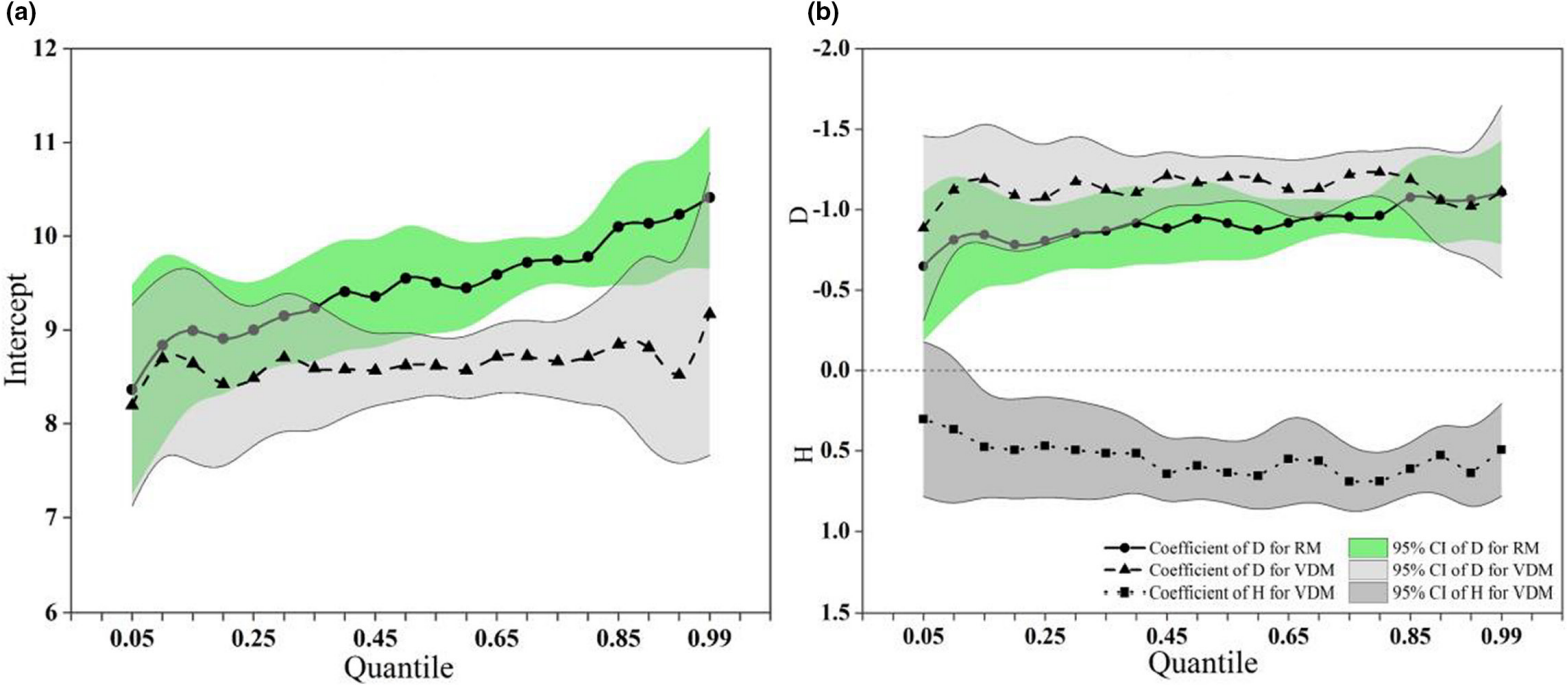
Model coefficients and their 95% confidence intervals of different quantiles for Reineke's model and the variable density model. (a) is the coefficient of intercept. (b) is the coefficient of *D* and *H*. (RM is Reineke's model, and VDM is the variable density model).

Reineke's model and the variable density model with different quantiles were fitted using quantile regression, and the trajectories of the slope and intercept are shown in Figure 4. The 95% confidence interval for each coefficient shows that the *D* of Reineke's model and the *D* and $H_{d}$ of the variable density model were statistically significant (except $H_{d}$ when τ<0.1). The stand density was negatively correlated with the independent variable *D* and positively correlated with the independent variable $H_{d}$ (Figure 4b). When τ≥0.90, the coefficient of *D* for both Reineke's model and the variable density model tended to be stable, which indicates that the size of the effect of *D* of the two models was similar. The coefficients of *D* were all significantly different from zero and significantly different from −1.605.

We fitted the self‐thinning models using stochastic frontier analysis. The model coefficients of Reineke's model and the variable density model were statistically significant (*p* < .05) (Table 3). The variable density model had a smaller AIC value and differed significantly from Reineke's model in terms of the goodness of fit (*p* < .001) for the three stochastic frontier analysis models (NH, NE, and NT). Compared with the variable density model for NH and NE, the NT model had a smaller AIC value. These results demonstrate that the variable density model was more suitable than Reineke's model for fitting the self‐thinning line.

**TABLE 3:** Parameter and variance estimates of Reineke's model (RM) and the variable density model (VDM) fitted by using stochastic frontier analysis (LRT correspond to the likelihood ratio test between Reineke's model and the variable density model)

Model	Variable	Intercept	Slope (*D*)	Slope (*H*)	$\sigma_{v}^{2}$	$\sigma_{u}^{2}$	AIC	LRT
NH‐RM	D	9.03 (0.744)	−0.732 (0.076)		0.381	0.125	177.4	<0.001
NH‐VDM	D, H	8.343 (0.370)	−1.011 (0.145)	0.631 (0.163)	0.302	0.371	138.8	
NE‐RM	D	8.956 (0.521)	−0.732 (0.130)		0.388	0.026	177.4	<0.001
NE‐VDM	D, H	8.253 (0.368)	−1.027 (0.146)	0.631 (0.167)	0.337	0.168	134.2	
NT‐RM	D	9.860 (0.315)	−0.738 (0.136)		0.372	0.058	173.4	<0.001
NT‐VDM	D, H	8.424 (0.542)	−0.996 (0.162)	0.621 (0.169)	0.301	0.378	130.6	

### 3.2 Evaluation of the statistical approach

**FIGURE 5:**
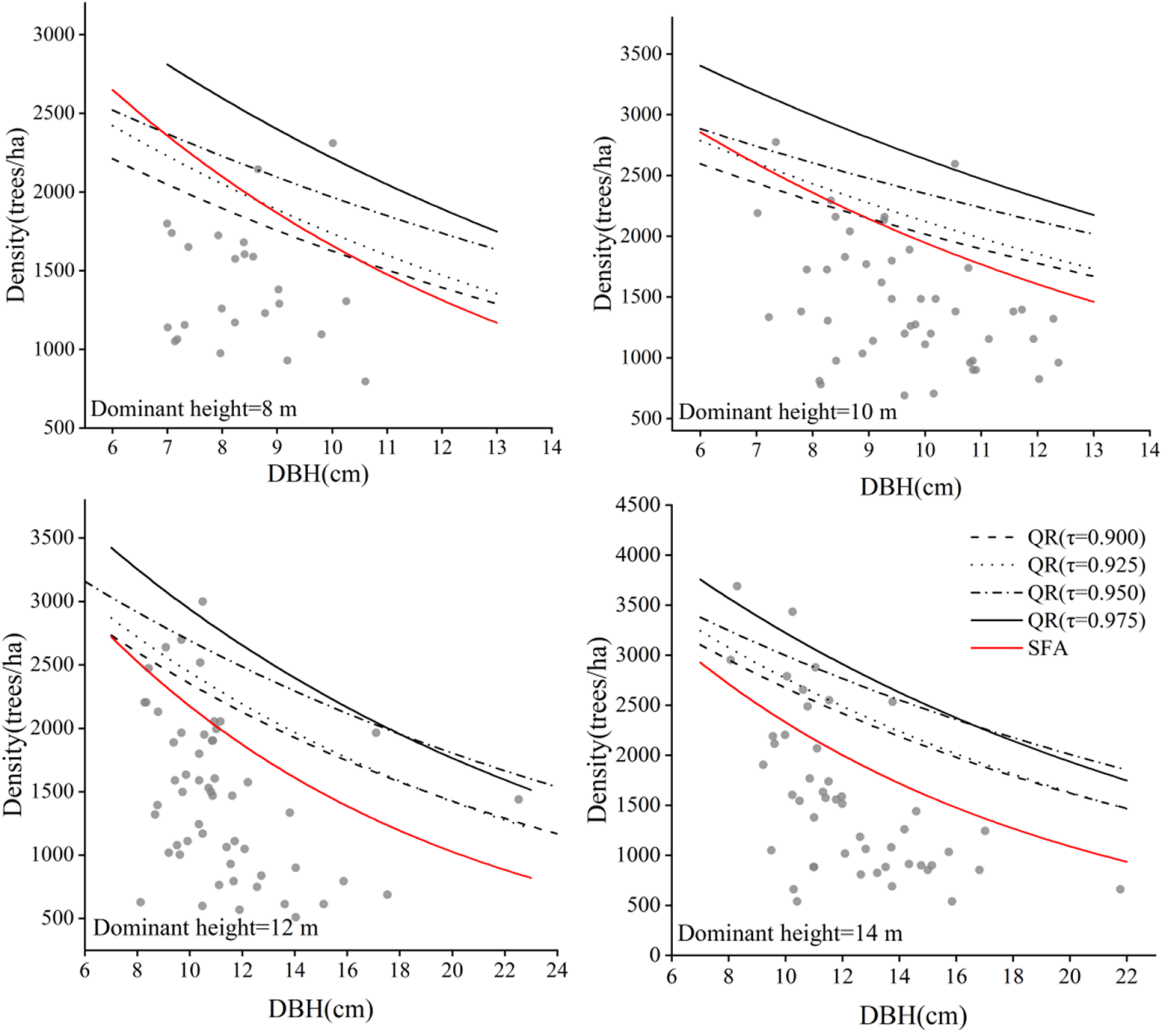
Maximum density line reflecting the relationship between stand density and stand average diameter based on specific tree height (QR is the quantile regression, *τ* is the quantile value, and SFA is the stochastic frontier analysis).

We fitted the variable density model using quantile regression (τ = 0.900, 0.925, 0.950, 0.975, 0.990) and stochastic frontier analysis separately. During parameter estimation, convergence was easily reached for the two statistical approaches, but the model with τ = 0.990 did not converge. The maximum density line with specified tree height is shown in Figure 5. With the increase in the DBH, the maximum carrying capacity showed a downward trend for all the maximum density lines. For quantile regression, different quantile values correspond to different curve slopes. It is difficult to decide which quantile can best reflect the maximum density of a stand. In contrast, the trend of the maximum density line fitted by stochastic frontier analysis was closer to the actual stand density.

**FIGURE 6:**
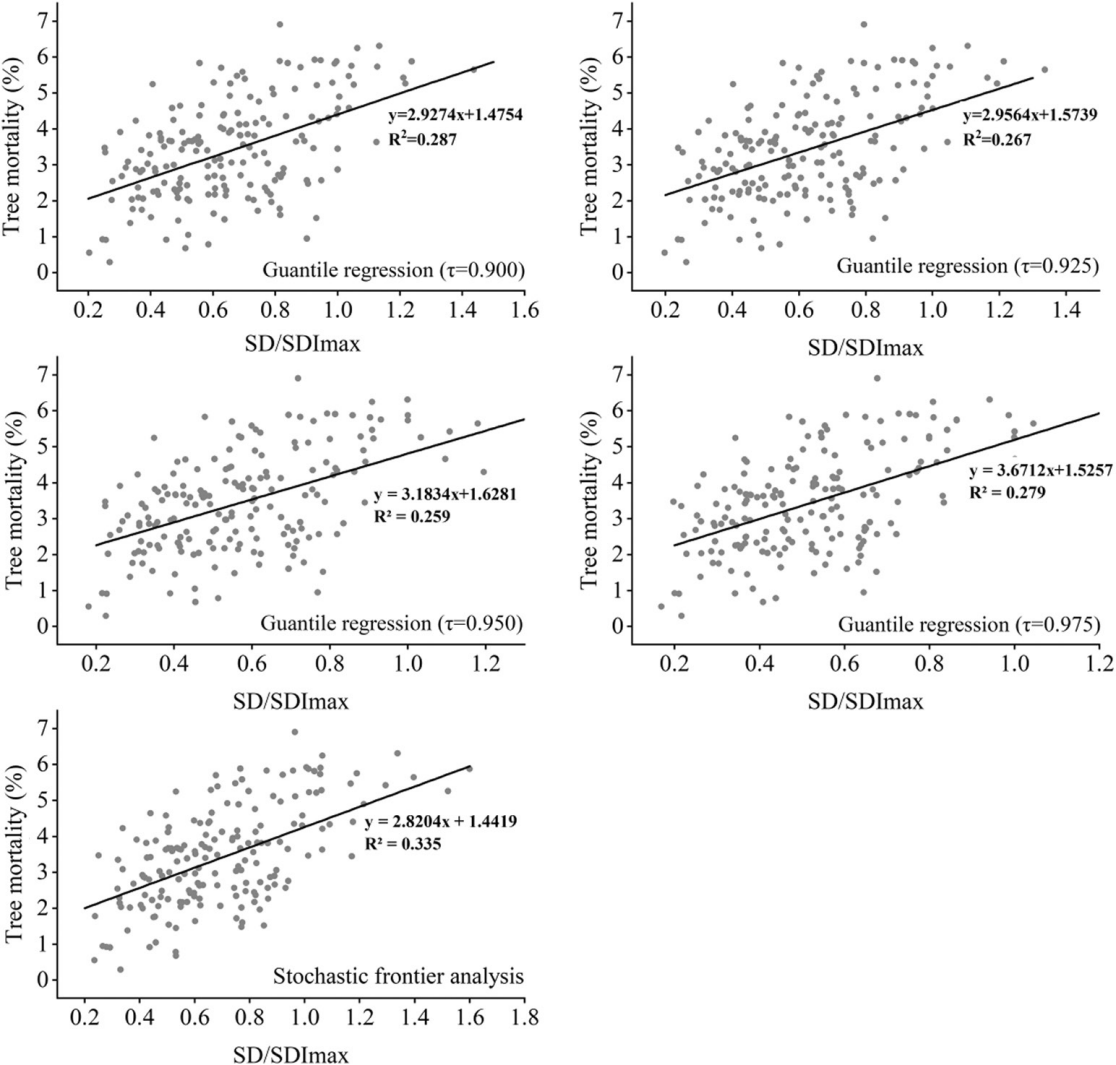
Tree mortality and SD/SDI_max_ showed a significant linear relationship in the statistical model estimated by quantile regression (τ = 0.900, 0.925, 0.950, 0.975, 0.990) and stochastic frontier analysis.

In addition, the relationship between tree mortality and SD/SDI_max_ (SD was the actual stand density) was assessed by using a linear function to evaluate the quantile regression and stochastic frontier analysis (Figure 6). Obviously, the higher the value of SD/SDI_max_ is, the higher the tree mortality. The fitting accuracy of quantile regression did not show obvious regularity with the increase in quantile value. The t test of residuals showed that the R^2^ of the model fitted by stochastic frontier analysis was significantly higher than that of the quantile regression function.

The validation samples were fitted using stochastic frontier analysis. We used a t test to evaluate the difference between the SDI_max_ of the validation samples and modeling samples, which was calculated according to Equation (12). The results showed that there was no significant difference between the average values of the SDI_max_ (*p* < .05). This demonstrated that the variable density model constructed with a stochastic frontier was reliable and stable.

### 3.3 Topography, stand structure, and density

**FIGURE 7:**
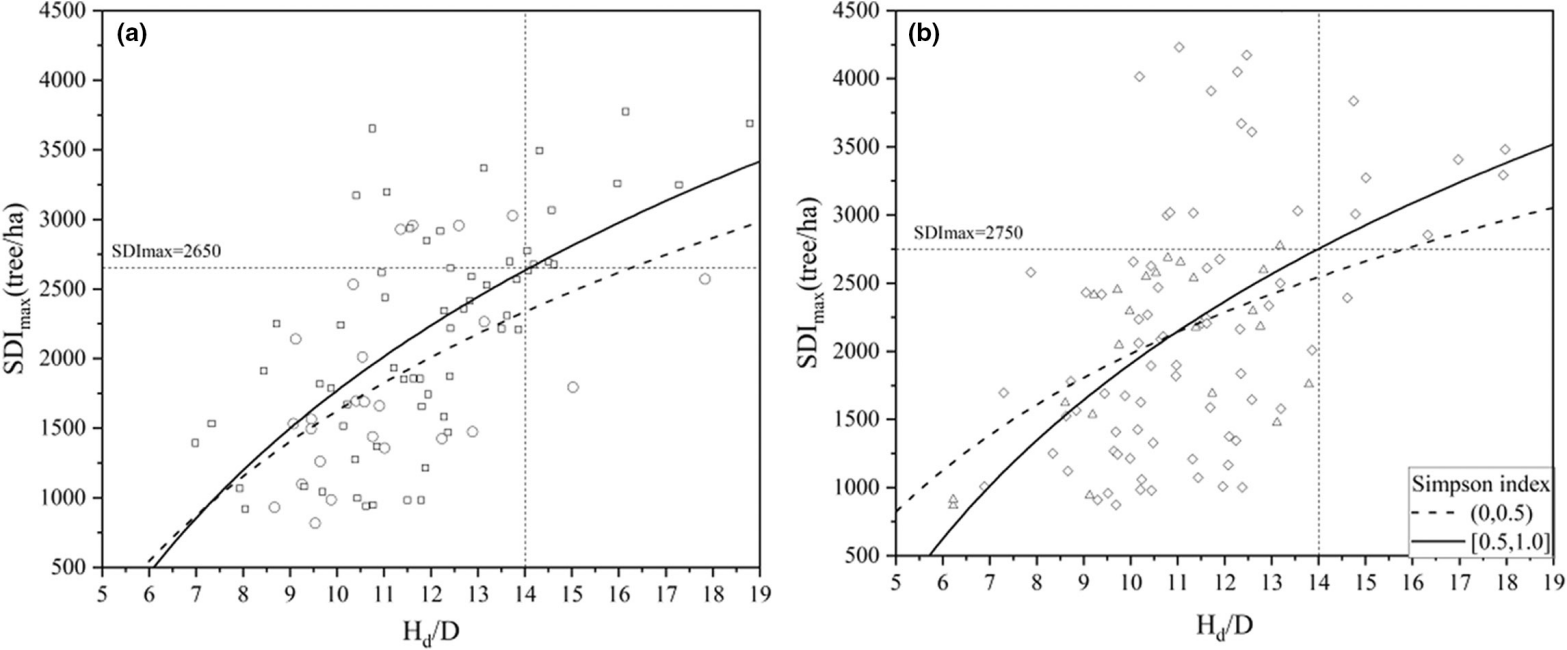
The relationship between SDI_max_ and *H*_d_/*D* in fixed values of the altitude and Simpson index. The altitude at 0–500 m was set to low (a), and the altitude at 501–1000 m was set to high (b). Simpson index levels were set to low (0–0.5) and high (0.5–1.0).

Based on a variety of alternative models, SDI_max_ was found to be effectively modeled as a function of altitude, Simpson index, and *H*_d_/*D*. The model had an error of approximately 25.8% with respect to the mean observed value of SDI_max_. The relationship between SDI_max_ and *H*_d_/*D* is shown in Figure 7 by using fixed values of the altitude and Simpson index. The results suggested that the SDI_max_ was sensitive to *H*_d_/*D*, which showed positive relationships with SDI_max_. Furthermore, the SDI_max_ had a slight positive relationship with altitude and the Simpson index in a specific interval.

## 4 DISCUSSION

### 4.1 Effect of the dominant height on density in mixed forests

Zeide (1985) attempted to modify Reineke's model and proposed a modified SDI equation: lnN=a−blnD+xHlnD, but the results showed that the inclusion of height did not decrease the model error. In our assessment, the inclusion of the dominant height was able to improve the model error because the variable density model fitted by stochastic frontier analysis showed a better goodness of fit than Reineke's model (Table 3). The results demonstrate that the effect of height on the density in the mixed forests was different from that in plantation forests.

Interestingly, the independent variable DBH in Reineke's model and the variable density model was negatively correlated with density (Table 3), but the independent variable H_d_ was positively correlated with density. This result indicates that the inclusion of the dominant height did not affect the self‐thinning rate but did affect the maximum stand carrying capacity in the mixed forests. When the dominant height increases, the number of canopy levels may increase, which may lead to an increase in the stand density (Figure 2c). However, situation 1 shown in Figure 2b does not hold because the dominant height did not affect the self‐thinning rate, and the competition did not decrease.

Stands with different dominant heights have different maximum stand carrying capacities; once the actual stand density exceeds the carrying capacity, self‐thinning begins. The advantage of the variable density model with dominant height is that the model can predict the maximum stand density and self‐thinning line in stands at various growth stages (Figure 5). Therefore, we recommend fitting the self‐thinning line using the variable density model in mixed forests because the measurement of the height does not increase the cost of applying remote sensing technology.

### 4.2 Suitable statistical methods to fit the self‐thinning model

Although quantile regression and stochastic frontier analysis have become common and preferred methods to fit the self‐thinning line, traditional methods remain important and indispensable. The self‐thinning model estimated by traditional methods is based on stand growth dynamics and has biological significance, in contrast to quantile regression and stochastic frontier analysis.

**FIGURE 8:**
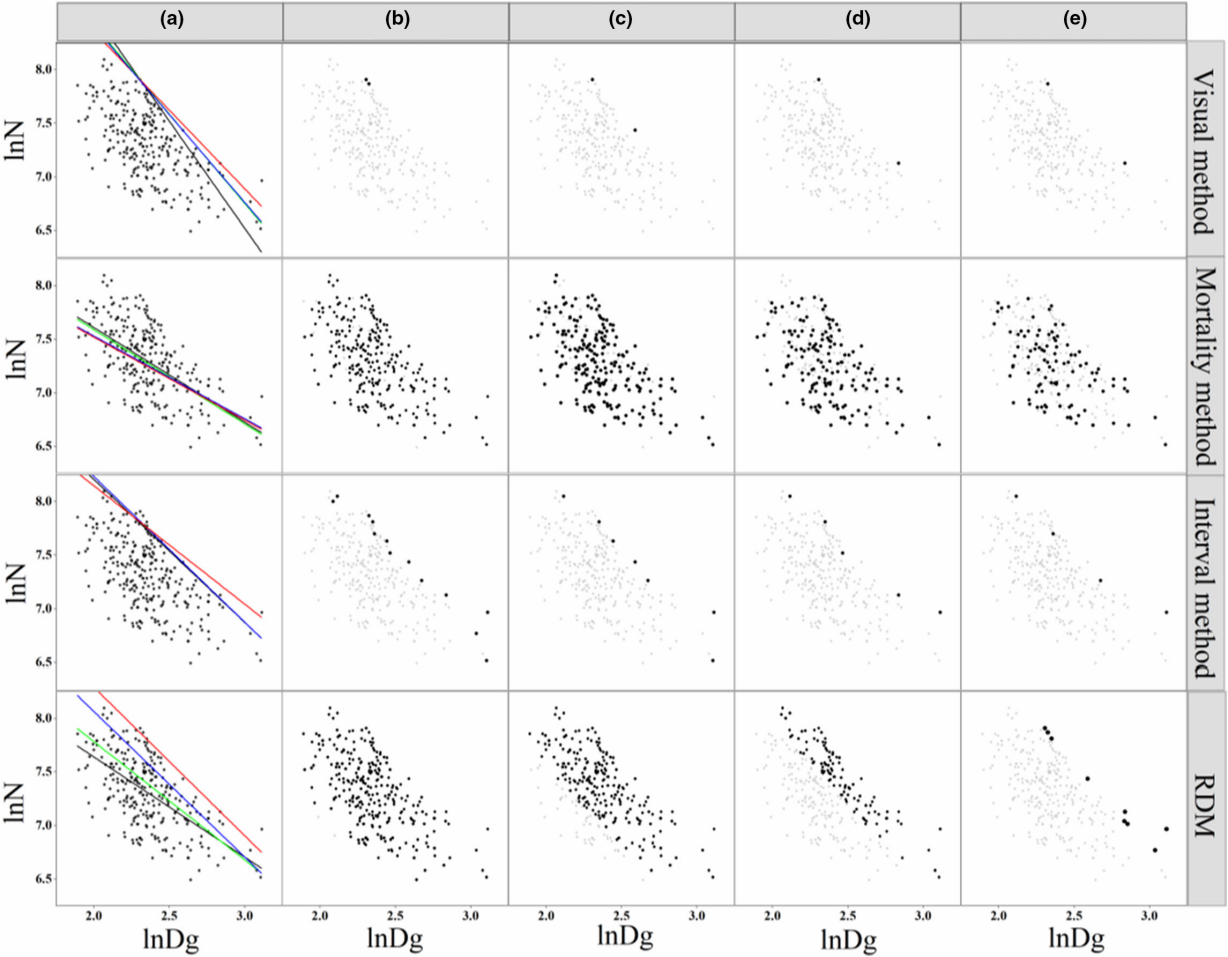
The data points selected using the four methods based on different standards and the schematic of the self‐thinning lines. (a) Different colored lines represent different self‐thinning lines; (b–e) different standards for the four methods; RDM is the relative density method.

Therefore, we also used traditional methods (visual method, mortality method, interval method, and relative density method) to fit the self‐thinning model of mixed forest (Figure 8). Westoby (1984) found that plantation forests began to self‐thin when the mortality reached 20% because tree death was the result of a high density. However, many tree deaths in mixed forests are the result of poor light and limited growth space, and the stand density is not the only reason for mortality. Therefore, the mortality method should be modified to make it suitable for mixed forests, which was confirmed in our study (Figure 8). Rational selection of the relative density standard is the key to modeling the self‐thinning line (Solomon & Zhang, 2002), and the self‐thinning line within the relative density standard above 1.0 was close to the upper edge of the stand density and showed a suitable goodness of fit in this study (Figure 8). We found that the interval method was able to avoid the disadvantages of the visual method, mortality method, and relative density method (Figure 8). However, it is possible to include some plots at lower densities that have not reached the stage of self‐thinning (Zhang et al., 2005). Consequently, the slope coefficient of the self‐thinning line based on this subset of the plots may be flatter than expected (Osawa & Allen, 1993; Westoby, 1984). Overall, the interval method was the most suitable for modeling the self‐thinning line among the four traditional methods.

Although quantile regression and stochastic frontier analysis can serve the same purpose of boundary delineation in applied statistics far beyond econometrics, an adequate appreciation of the differences between the two approaches is essential for researchers to select the best method to estimate the self‐thinning surface (Tian et al., 2020). The value of τ chosen for estimating the self‐thinning line mainly ranges from 0.90 to 0.99 in the literature, with 0.95 ≤ τ ≤ 0.99 being the most common choice (Andrews et al., 2018; Condés et al., 2017; Vospernik & Sterba, 2015). However, without a careful comparison to strike a balance between the quantiles, quantile regression is likely to introduce a certain degree of subjectivity. Compared with the quantile regression, we found that the stochastic frontier analysis was superior in model prediction (Figure 6). Furthermore, stochastic frontier analysis may lead to a more objective self‐thinning line because it does not involve the subjective selection of a particular value of τ. However, quantile regression can still serve as a valuable complement to stochastic frontier analysis in the estimation of the self‐thinning surface, as it allows the impact of variables other than stand density on different quantiles to be examined (Tian et al., 2020).

### 4.3 Effect of topography and structure on density in mixed forests

The significant correlations between the stand density and the site quality and climate have been confirmed in the literature (Long & Shaw, 2005; Zhang et al., 2013). Site quality has a significant influence on the intercept of the self‐thinning line; the better the site quality is, the higher the carrying capacity (Kimsey et al., 2019; Weiskittel et al., 2009). Generally, the stand density should be higher in areas with low altitudes and slopes due to the negative correlation between the site quality and the altitude and slope. In contrast, a higher density was found at higher altitudes and slopes in this study (Figure 7). There are two possible reasons for this finding: (1) Site quality is likely to be the result of a combination of factors (de Prado et al., 2020; Weiskittel et al., 2009), not only altitude and slope; and (2) there is a high population density in the areas with low altitudes and slopes, and frequent anthropogenic activities may result in a decrease in the stand density.

The stand structure (e.g., tree species diversity) has significant effects on the stand density and tree competition (Weiskittel et al., 2009). Unfortunately, significant effects of tree species diversity on stand density were not found in this study (Figure 7), but the higher the distribution uniformity of tree species was, the higher the stand density. Generally, in a stand, a higher distribution uniformity of tree species will lead to a higher overlap and a lower competition and mortality, which is one of the reasons for the increase in stand density.

## 5 CONCLUSION

The stochastic frontier analysis was superior to quantile regression in the model prediction and objective evaluation. The proposed variable density model exhibited a better goodness of fit and residual distribution than Reineke's model for modeling the self‐thinning line for oak mixed forests. The coefficients of the quadratic mean diameter and dominant height in the variable density model represent the self‐thinning rate and the variable maximum stand carrying capacity, respectively; the interpretation of the coefficients makes this approach more biologically relevant than Reineke's model. The stand density in the oak mixed forests was influenced significantly by the altitude, Simpson index, and $H_{d}$/*D*.

## AUTHOR CONTRIBUTIONS

**Shisheng Long:** Conceptualization (lead); data curation (lead); formal analysis (lead); investigation (lead); methodology (lead); resources (equal); software (lead); supervision (equal); validation (equal); visualization (lead); writing – original draft (lead); writing – review and editing (lead). **Siqi Zeng:** Conceptualization (equal); data curation (equal); formal analysis (equal); funding acquisition (lead); project administration (lead); resources (equal); writing – original draft (equal); writing – review and editing (equal). **Zhenwei Shi:** Data curation (equal); investigation (equal); methodology (equal); resources (equal); supervision (equal); visualization (equal). **Shengyang Yang:** Data curation (equal); investigation (equal); software (equal); validation (equal); visualization (equal).

## CONFLICT OF INTEREST

The authors declare that they have no known competing financial interests or personal relationships.

## Data Availability

The data presented in this study have been uploaded to Dryad (https://doi.org/10.5061/dryad.931zcrjnq).
